# Ecofriendly and smart spectrophotometric approaches for synchronized analysis of two antipsychotic drugs, fluoxetine and olanzapine: application to combined tablet dosages with assessment of method greenness

**DOI:** 10.1186/s13065-025-01398-1

**Published:** 2025-02-13

**Authors:** Sayed M Derayea, Huda Madian, Ebtihal Samir, Khaled M. Badr-eldin

**Affiliations:** 1 Analytical Chemistry Department, Faculty of Pharmacy, Minia University, Minia, 61519 Egypt; 2 Pharmaceutical Analytical Chemistry Department, Deraya University, New Minia, 61519 Minia Egypt

**Keywords:** Fluoxetine, Olanzapine, Ratio subtraction method, Dual wavelength method, Absorptivity factor method, Combined pharmaceutical dosage forms

## Abstract

The present study was dedicated for solving the challenge of the overlapped spectra of olanzapine (OLZ) and fluoxetine (FLX) for their concurrent, accurate and precise determination without prior physical separation. The developed methods had to venture away from those that depend on chromatography, because of their negative effects on the environment due to using hazardous organic solvents as well as their high cost. The suggested methods were based on spectrophotometry combined with simple mathematical treatments of the spectra. These approaches are simple, rapid, inexpensive and reliable options for the effective assay of FLX and OLZ in pharmaceutical dosage forms. Ratio subtraction method was devoted for estimation of both drugs, while dual wavelength method and absorptivity factor method could be utilized in the estimation of FLX and OLZ, respectively. All the suggested methods were efficiently applied for the determination of the studied drugs in laboratory prepared mixtures and their pharmaceutical formulations. The environmental safety of the involved procedures was assessed by applying the Eco Score scale and complex modified GAPI methods. The methods were proved to be highly safe since no large volume of solvents were used, and no derivatizing reagent or drastic experimental conditions were involved. In addition, the procedures consume little energy and produce a small amount of waste. In conclusion, for FLX determination, the use of dual wavelength method is preferable because it needs very low mathematical treatment than the ratio subtraction method. However, for OLZ, the ratio subtraction method has higher sensitivity than absorptivity factor method although the latter is simpler.

## Introduction

Depression affects more than 400 million people worldwide and, according to statistics, almost 20% of the world’s population will experience at least one episode of depression throughout their lives. Of these cases, almost half will have repeated attacks and need continuous treatment. Therefore, the World Health Organization (WHO) assumed that in 20 years depression will become the second most expensive and fatal disease.

Fluoxetine (FLX) selectively inhibits serotonin reuptake and is widely prescribed for treating depression and panic disorders. It belongs to second generation antidepressant which is more acceptable in terms of tolerance and toxicity [1]. Olanzapine (OLZ) is an atypical antipsychotic, which belongs to the thioenobenzodiazepine group. It is used in the treatment of schizophrenia and other related syndromes [2]. It is more effective than other psychotropics in the management of type I bipolar disorder, because it produces better response without the side effects presented by other antipsychotics.

Cases involving depression associated with bipolar disorder are even more serious, as the patient goes through states of extreme euphoria, mania and deep depression. An effective and widely prescribed treatment for such cases is the combination of FLX and the atypical antipsychotic OLZ, which act synergistically by increasing the levels of the three main neurotransmitters (serotonin, dopamine and noradrenaline). Low levels of these neurotransmitters can cause depression. The action of the two drugs together is more effective with lower side effects compared to any of the components alone [3].

Based on their therapeutical importance for treating cases involving depression associated with bipolar disorder, combinations of FLX and OLZ, were simultaneously analyzed using various analytical methods. The published methods included UV spectrophotometry [4–6], TLC [6–9], HPLC [6, 8–16], and LC–MS [17–22]. However, there are several limitations in HPLC methods such the use of mobile phases that contain various organic solvents and modifying agents, possibility for precipitate formation because of using mobile phase that contains ammonium salts in presence of methanol or acetonitrile, high consumption of hazardous organic solvents, and long analysis time. Furthermore, some of the reported HPLC methods were extremely expensive owing to the use of MS/MS detectors. This invoked the development of greener methods that could effectively overcome these limitations and offer rapid and straightforward determination of the two drugs simultaneously with low efforts.

The use of spectrophotometry hyphenated with chemometric approaches is a powerful tools for resolving spectral overlap, allowing the concomitant determination of several drug combinations without the need of physical separation before the analysis [23]. Most of these methods do not require expensive equipment or certain training or special software. Furthermore, there are a variety of computational methods from which the analyst can select what suits his purpose.

The present study aimed to develop straightforward spectrophotometric methods combined with simple mathematical treatments that could solve the severely overlapped spectra of FLX and OLZ. Three different mathematical approaches were utilized which included: dual wavelengths, ratio subtraction and absorptivity factor methods. The suggested approaches have simple and fast procedures, and could be utilized for the accurate and precise assay of marketed formulations containing the drugs combination. The methods were assessed in respect to ICH rules [24] and their results were compared to evaluate the effectiveness of these methods.

## Principle and theoretical calculations

### Ratio subtraction method (method I) [25–27]

This method only needs the presence of a plateau region in the ratio spectrum. If there are two analytes A and B with overlapping spectra, the concentration of drug having this feature (for example A) can be estimated by employing the method of ratio subtraction. The first step is the division of the spectrum of the solution containing mixture of both analytes by the spectrum (divisor, B`) of a suitable concentration of B. A plateau region (constant absorbance) should appear in a region corresponding to analyte (B). The equation representing the division of the spectrum of the mixture by the spectrum B could be expressed as follow: -

$$\frac{(A+B)}{{B}^{\prime }}=\frac{A}{{B}^{\prime }}+\frac{B}{{B}^{\prime }}=\,\frac{A}{{B}^{\prime }}+\,constant$$

The next step is to subtract the constant from the ratio spectrum then multiplied by the same divisor (B’). The obtained spectrum would correspond to the original spectrum of drug A. Finally, the concentration of drug (A) can be estimated using the linear regression equation of the pure drug at its λ_max_. The concentration of drug (B) can be obtained using the same method if it exhibited this feature or using any other suitable method.

### Dual wavelength method (method II ) [26, 28]

In this method, one of the two drugs was considered as the analyte while the other drug is treated as an interfering substance. Thus, two wavelengths were selected so as the interfering substance should have the same absorbance values, whereas the analyte should exhibit the greatest difference in absorbance that has direct proportionality with the analyte concentration and do not depend completely on the concentration of the interference substance. The choice of the two wavelengths is very important to achieve the highest selectivity and sensitivity.

### Absorptivity factor method (method III) [26]

If the spectra of two overlapping drugs having equal concentrations do not have any point of intersection, then iso-absorptive point cannot be applied. An alternative and effective method to resolve such spectral overlap is the use of a similar approach called absorptivity factor method. In this method, the concentration of one drug (A) is doubled that of the other drug (B) so as to obtain a point of intersection between their zero spectra. In this case the absorptivity of drug (A) would be one half that of drug (B) at the intersection wavelength (λ). At this λ: 2 ε_(A)_ = ε_(B) =_ ε, where ε is the absorptivity using 1.0 molar concentration and 1 cm as path length. The absorbance of the mixture (A_M_) of the two drugs, will correspond to the concentrations of both drugs according to following equation:

$$ {{\rm{A}}_{\rm{M}}}\,{\rm{ = }}\,{\rm{\varepsilon }}\left( {{\rm{2}}{{\rm{C}}_{{\rm{AM}}}}{\rm{ + }}{{\rm{C}}_{{\rm{BM}}}}} \right) $$

Where the concentrations of the individual drug A and B in the mixture are C_AM_ and C_BM_, respectively. If the concentration of one of the two drugs could be obtained by another method, then, the concentration of the other drug can be obtained by simple subtraction.

## Experimental

### Instrumentation

The spectrophotometric measurements were carried out on a spectrophotometer made by Shimadzu UV-Visible 1900i (Tokyo, Japan). The device was controlled with software installed in PC and two quartz cuvettes (1 cm) were used, one for the blank solution and the other for the tested solution.

### Chemical and reagents

Analytical grade chemicals and solvents were used throughout the present work. Pure powders of FLX (99.5% purity) and OLZ (99.8% purity) were supplied by Egyptian International Pharmaceutical Industry (EIPICO, 10th of Ramadan City, Cairo, Egypt). The commercial pharmaceutical formulations were purchased from local Egyptian pharmacies. Flunzapine 6/25 mg^®^ capsules (containing 6 mg FLX and 25 mg OLZ per capsule) were produced by Delta Pharma (10th of Ramadan City, Cairo, Egypt). Raibyax 12/25 mg^®^ capsule (containing 12 mg FLX and 25 mg OLZ per capsule) is a product of Rameda for Pharmaceutical Industry (2nd Industrial Zone, 6th October, Egypt). Solvents were brought from El-Nasr Chemical Company (Cairo, Egypt).

### Standard solutions of FLX and OLZ

The stock solutions of the individual drugs were prepared by weighing accurately 10.0 mg of the pure powder of either drug and dissolving in 100 ml calibrated flask using 0.1 N HCl. The volume was completed to 100 mL to give100,0 µg mL^− 1^ solution. The solutions should be kept in refrigerator till the time of analysis.

### Procedures

#### General assay procedures

##### Method I (ratio subtraction)

Calibration curves for the pure individual drugs were built up at each specific λ_max_ i.e. at λ_max_ 262.2 nm for FLX and 258 nm for OLZ. A series of standard solutions of each drug (4.0–24.0 µg mL^− 1^ for FLX or 2.0–12.0 µg mL^− 1^ for OLZ) were made and the absorbance at the specific λ_max_ of each was correlated with its concentration. For determination of OLZ, the spectrum of the mixture containing both drugs was scanned and divided by a divisor which was the spectrum of a fixed concentration of FLX (24 µg mL^− 1^). The constant value at 290 nm was subtracted from the ratio spectrum. The resulting spectrum is then multiplied with the divisor spectrum to resolve the original spectrum of OLZ. The concentration of OLZ could be directly retrieved from the absorbance at λ_max_ of the resolved spectrum. The same sequence could be followed for obtaining the resolved spectrum of FLX from the mixture using 15 µg mL^− 1^ as divisor and the constant value was at 220 nm in the ratio spectrum.

##### Method II (dual wavelength method)

The dual wavelength method was utilized for estimating FLX in presence of OLZ. The calibration graph of FLX was constructed using its pure standard solutions (4.0–25.0 µg mL^− 1^). The absorbance at both 226.2 nm and 280 nm were recorded and their difference was estimated (ΔA_FLX_ = A_226.2 nm_– A_280 nm_. The absorbance difference for OLZ at these two wavelengths was zero. Then, the obtained ΔA_FLX_ values were plotted against the corresponding FLX concentration.

##### Method III (absorptivity factor method)

Solutions of FLX (20.0 µg mL^− 1^), OLZ (10.0 µg mL^− 1^) and their mixture containing 10.0 µg mL^− 1^ FLX and 5.0 µg mL^− 1^ OLZ were prepared. The zero-order spectrum of each solution was recorded. The calibration graph of OLZ was set up by plotting the drug concentration of a series of standard solutions (2.0–12.0 µg mL^− 1^) versus their absorbance at 231.2 nm. The values of the absorbance of mixtures containing the two drugs were also recorded at 231.2 nm. The obtained absorbance was utilized for estimating the total concentration of both drugs in the mixture using linear regression equation of OLZ at 231.2 nm. The concentration of FLX in the mixture was estimated using the dual wavelength method (Method II) by measuring the absorbance at 226.2 and 280 nm as previously described, then OLZ concentration was estimated by subtraction.

#### Procedure for analyzing laboratory prepared mixtures

Mixtures of the drugs solutions were prepared in different ratios (1:2, 1:2.1. 1:4.2, 1:8.3 of FLX and OLZ respectively) by mixing the suitable portions of FLX and OLZ standard solutions (100 µg mL^− 1^) in a series of 10 mL calibrated flasks. Each solution was finalized to 10 mL with 0.1 N HCl and mixed thoroughly. The assay was performed as previously described.

#### Procedure for analyzing pharmaceutical dosage forms

Ten capsules (Flunzapine 6/25 mg^®^ or Raibyax 12/25 mg^®^ capsules) were evacuated, their contents were mixed thoroughly and weighed accurately. An accurate weight of the mixed powdered capsule corresponding to 100.0 mg of FLX and 48 or 24 mg of OLZ was moved to 100 mL volumetric flask. The powder was extracted with about 50 mL of 0.1 N HCl, by sonication for 15 min. Additional volume of the same solvent was added to complete the flask to the final volume. The flask contents were thoroughly mixed followed by filtration to remove the insoluble ingredients. After removing the initial filtrate, a portion of the filtrate was further diluted with 0.1 N HCl to get solution having a final concentration of 25/6–25/12 µg mL^− 1^ of FLX/ OLZ, respectively. An aliquot of the final solution was analyzed using the analytical procedures described previously to determine the concentrations of the FLX and OLZ.

## Results and discussion

Combined dosage forms containing two antipsychotics; OLZ and FLX are widely utilized in treating bipolar depression. The direct simultaneous spectrophotometric measurements of both drugs were not possible due to the strong overlap in their UV spectra. The use of spectrophotometry coupled with mathematical approaches was selected to offer simple, rapid, green, inexpensive, and cost-effective, as well as reliable ways for the concurrent assay of OLZ and FLX in laboratory mixtures and commercial dosage forms. No physical separation was needed before doing the analysis. The sample was directly measured after dissolution in the suitable solvent followed by simple mathematical treatment of the obtained spectra of the drugs mixtures. No special equipment, software or extensive training were required.

### Analytical methods

#### Ratio subtraction method

The zero-order absorption spectrum of a solution that contained both drugs was recorded in the range of 220–300 nm. The zero absorption spectra of the individual solutions containing 24 µg mL^− 1^ FLX and 15 µg mL^− 1^ OLZ were also recorded. For the assay of OLZ, the spectra of the mixture were divided by the spectrum of 24 µg mL^− 1^ FLX to give the ratio spectra (*Spec 1*) which could be represented as $\:\frac{\varvec{O}\varvec{L}\varvec{Z}}{\varvec{F}\varvec{L}\varvec{X}}+\varvec{c}\varvec{o}\varvec{n}\varvec{s}\varvec{t}\varvec{a}\varvec{n}\varvec{t}$. The value of this constant can be obtained from the plateau region in the ratio spectra from 220 to 230 nm. The value in ratio spectra at 220 nm was chosen as the constant and subtracted from the ratio spectra of the mixtures then the resulting spectra were multiplied by the spectrum of 24 µg mL^−1^ FLX. The final spectra correspond to the original OLZ spectra. In this case the concentration of OLZ could be estimated from the resolved spectra at the specific λ_max_ of OLZ (258 nm) by applying the regression equation driven using pure OLZ. The same steps can be carried out for resolving the original spectra of FLX but in this case, the spectra of the mixtures were divided by the spectrum of 17.5 µg mL^− 1^ OLZ and the selected constant value was at 290 nm (plateau region at 280–300 nm). The concentration of FLX can be obtained using the absorbance value of the resolved spectra at its λ_max_ of 226 nm.

#### Dual wavelength method

**Fig. 1:**
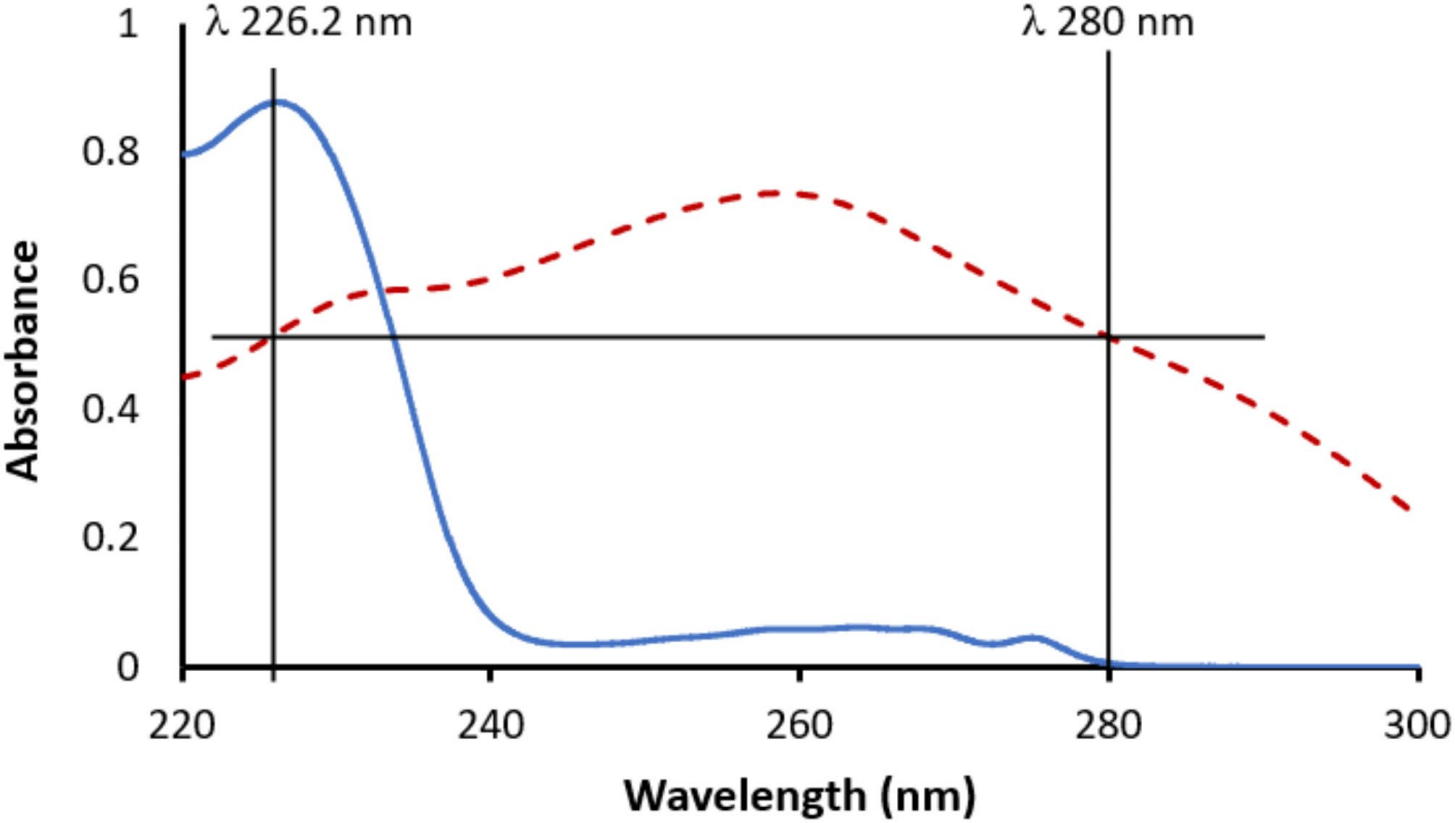
Absorption spectra of (___) 24 µg mL^-1^ FLX and (.......) 10 µg mL^-1^ OLZ showing the dual wavelength for determination of FLX

The spectra of both OLZ and FLX were carefully inspected to find two wavelengths at which one component shows the highest difference in absorbance while the other drug shows equal absorbance. As shown in Fig. 1, it was clear that 226.2 nm and 280 nm could fulfill this task. At the two selected wavelengths, OLZ absorbance was the same (ΔA_OLZ_= zero) while FLX absorbance exhibited the highest numerical difference, and the absorbance difference of FLX was directly dependent on its concentration. After that, the calibration graph was constructed using standard solution of FLX alone. and the absorbance difference at both wavelengths (A_226.2 nm_– A_280 nm_) was graphed versus FLX concentration. The calibration was also examined in the presence of a fixed OLZ concentration, and it was found that no difference from that for pure FLX. Thus, the concentration of FLX in the mixture can be obtained from the direct measurement of the absorbance at the selected wavelengths without any interference from OLZ.

#### Absorptivity factor *method*

**Fig. 2:**
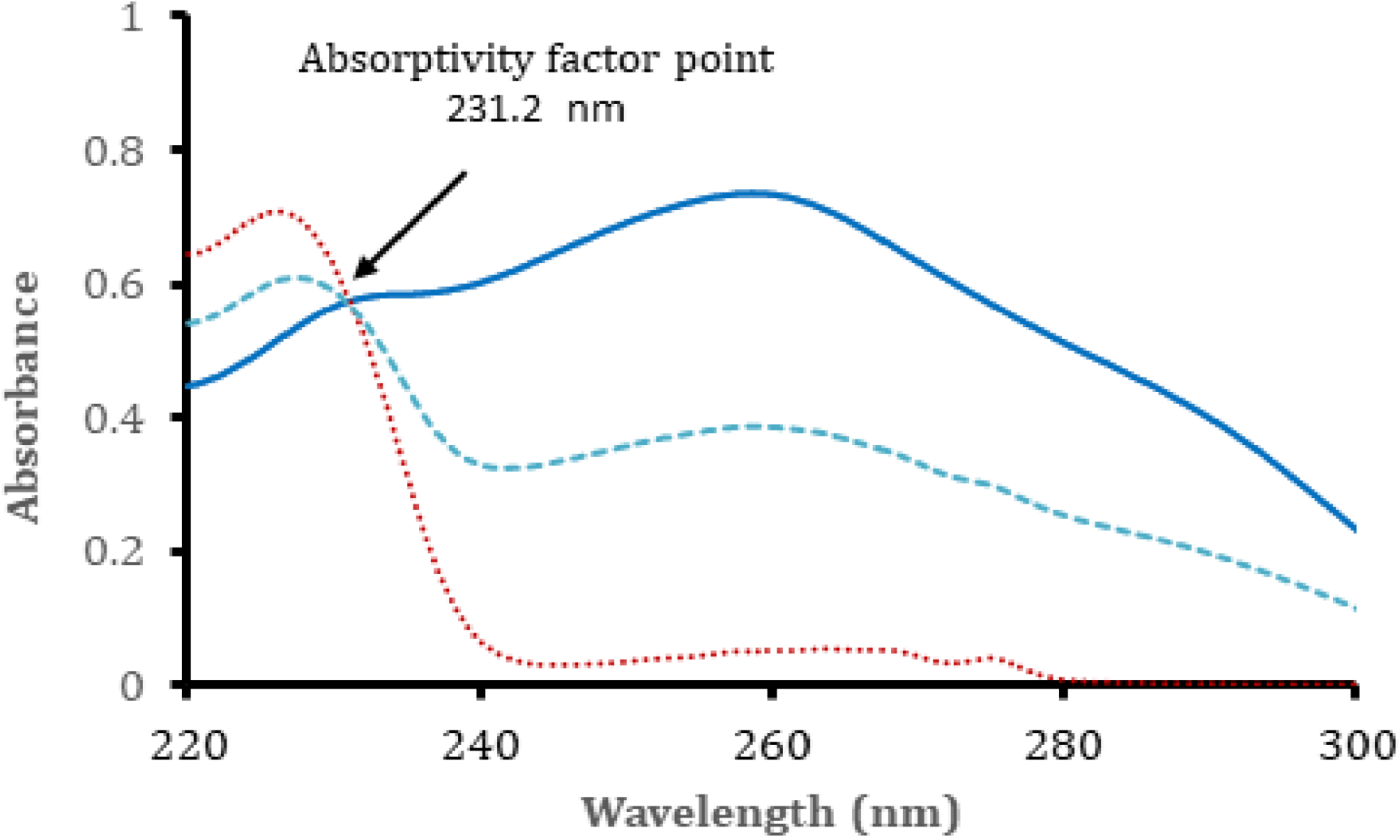
Zero order spectra of (**—**) 10.0 µg mL^-1^ OLZ, (**.... **) 20.0 µg mL^-1^ FLX and (- - - -) mixture containing 5 µg mL^-1^ of OLZ and 10.0 µg mL^-1^ FLX, showing the absorptivity factor wavelengths at 231.2 nm

The absorption spectra of both FLX and OLZ do not show any point of intersection if equal concentration were used. Therefore, the use of iso-absorptivity point method could not be applied. Another similar mathematical approach could be applied if a point of intersection could be present when the concentration of one of the two drugs is doubled. This method is called absorptivity factor method. When the concentration of FLX is two folds of that of OLZ, the overlapped spectra of both drugs show a point of intersection at 231.2 nm. At this point, the absorptivity of OLZ is equal to double that of FLX. Figure 2 illustrates the absorption spectra of 10.0 µg mL^− 1^ of OLZ, 20.0 µg mL^− 1^ of FLX and a solution containing 10.0 µg mL^− 1^ of OLZ and 5 µg mL^− 1^ of FLX showing the intersection point at 231.2 nm. The concentration of FLX was obtained with other methods (ratio subtraction or dual wavelength methods). Then OLZ concentration can be computed by subtracting FLX concentration (divided by 2) from the obtained concentration from the absorbance at the intersection point of the mixture.

### Validation of the proposed methods

The analytical methods validation was carried out as per ICH guiding rules including linear ranges, accuracy, precision, limits of detection and limits of quantitation [24].

#### Linear range, detection limit (LOD) and quantitation limit (LOQ)

The absorption spectra of the individual standard solution of the studied drugs were recorded. The analytical signal in each method for the specific drug was correlated with its concentration and the data were subjected to linear regression curve`s fitting. Calibration graphs of the proposed spectrophotometric method were constructed. The statistical parameters were computed and summarized in Table 1. The values of LOD and LOQ were computed based on the equations (LOD = 3.3 σ/S and LOQ 10 σ/S). The symbol σ and S represent the standard deviation of intercept and slope of calibration curve, respectively.

**Table 1:** The statistical parameters of the proposed methods for determination of the studied drugs

Parameter	Ratio substruction		Dual λ	Abs. factor
	OLZ	FLX	FLX	OLZ
Linear range (µg mL^− 1^)	2–12	4–24	4–30	5–15
Slope	0.069	0.038	0.037	0.057
SD of slope (S_b_)	0.0004	0.0002	0.0002	0.0003
Intercept	0.0082	-0.0008	0.0024	0.0062
SD of the intercept (S_a_)	0.0029	0.0026	0.0036	0.0027
Correlation coefficient **r**^2^	0.9994	0.9996	0.9996	0.9998
Determination coefficient	0.9997	0.9998	0.9998	0.9998
SD of residuals (S_y, x_)	0.006	0.006	0.006	0.003
LOD (µg mL^− 1^)	0.14	0.23	0.32	0.16
LOQ (µg mL^− 1^)	0.41	0.68	0.99	0.48

^a^ Number of determinations is 6, LOD: Limit of detection, LOQ: Limit of quantitation

##### Ratio subtraction method (method I)

The absorbance values of FLX and OLZ at 226.2 and 258 nm, respectively were utilized in the setting their calibration graphs and linear regression equations were computed. Correlation coefficients of 0.9995 and 0.9999 in concentrations ranges of 4.0–24.0 and 2.0–12.0 µg mL^− 1^ were obtained for FLX and OLZ respectively (Table 1). The values of LOD were 0.23 and 0.14 µg mL^− 1^ while LOQ were 0.68 and 0.41 µg mL^− 1^ for the two drugs, respectively.

##### Dual wavelengths method (method II)

This method was employed for the determination of FLX only because its spectrum could fulfil the requirement for this method. However, the method could not be utilized for OLZ determination because there were no two points at which the absorbance difference is zero for OLZ while FLX shows significant difference. A strong correlation was found between ΔA (A_226.2 nm_– A_280 nm_) of FLX (*r* = 0.9996) in the drug concentrations ranging from 4.0 to 30.0 µg mL^− 1^. The value of LOD was 0.32 µg mL^− 1^and LOQ was 0.99 µg mL^− 1^, which are slightly higher than the ratio subtraction method.

##### Absorptive factor method (Method III)

The absorbances of varied concentrations of OLZ were recorded at 231.2 nm (at which the absorptivity of OLZ is double that for FLX). The obtained absorbance values were utilized in the linear regression analysis. The method exhibited high linearity (*r* = 0.9998) in range of concentrations of 5.0–15.0 µg mL^− 1^. LOD was 0.16 µg mL^− 1 \^and LOQ was 0.48 µg mL^− 1^. These values are slightly higher than that of ratio subtraction method.

#### Accuracy

Accuracy of the proposed methods was assessed through the determination of FLX and OLZ using 4 different concentration levels. Mixtures of both drugs with fixed ratio (2:1, FLX: OLZ) were prepared and subjected to drug analysis by the proposed methods. The suitable linear regression equation was employed to compute the concentration of the drug in the prepared mixture. Both the recovery percentage and standard deviation were computed and summarized in Table 2. The results indicated that the developed methods are highly accurate as the mean percentage recovery values were close to 100% with low scattering from the mean value.

**Table 2:** Assessment of the accuracy of the proposed chemometric spectrophotometric methods for the determination of **FLX** and **OLZ** at four concentration levels

Method	Drug	FLX: OLZ. (µg mL^− 1^)	Found ( µg mL^− 1^)	% Recovery ^a^ ± SD	RSD
**Ratio Subtract**	**OLZ**	8:4	4.01	100.31 ± 0.375	0.374
		12:6	6.08	101.46 ± 0.30	0.296
		16:8	7.99	99.94 ± 0.451	0.45
		20:10	9.88	98.85 ± 0.543	0.549
	**FLX**	8:4	8.07	100.85 ± 0.64	0.63
		12:6	12.07	100.59 ± 0.283	0.281
		16:8	15.84	98.97 ± 0.283	0.285
		20:10	19.97	99.83 ± 0.296	0.297
**Dual wavelengths**	**FLX**	12:6	12	100.01 ± 0.908	0.907
		16:8	16.07	100.46 ± 0.851	0.847
		20:10	19.99	99.98 ± 1.021	1.021
		24:12	23.94	99.78 ± 0.851	0.853
**Absorptivity factor**	**OLZ**	12:6	5.94	99.11 ± 1.26	1.27
		16:8	7.97	99.68 ± 1.15	1.16
		20:10	9.92	99.2 ± 0.335	0.338
		24:12	11.93	99.46 ± 1.191	1.197

^a^ the value is the average of three determinations, SD is the standard deviation and RSD is the relative standard deviation

#### Precision

Two levels were examined to assess the precision of the developed methods. Standard solutions having three varied concentrations of FLX and OLZ in three mixtures with constant ratio were prepared. The prepared mixtures were analyzed three times by the suggested methods in the same day to assess the intra-day precision. For assessment of the precision at inter-day level, the assay was carried out in three separate days. Each analysis was carried out in triplicates and both recovery percentage and relative standard deviation were computed. The values of RSD were taken as an indication of the precision level. As shown in Table 3, the RSD values were below 2% providing evidence about the high precision level of the suggested methods.

**Table 3:** Intra- and inter-day precision of the proposed chemometric spectrophotometric methods for the determination of **FLX** and **OLZ**

Method	Drug conc. (µg mL^− 1^)		Intra-day precision		Intra-day precision	
			% Recovery ^a^± SD	RSD	% Recovery^a^± SD	RSD
Ratio Subtract	OLZ	6	101.02 ± 1.01	1.00	100.96 ± 0.98	0.97
		8	100.58 ± 0.65	0.65	100.32 ± 0.96	0.95
		10	98.61 ± 0.41	0.42	98.95 ± 0.37	0.38
	FLX	8	100.37 ± 0.94	0.93	100.49 ± 0.84	0.83
		12	100.28 ± 0.70	0.69	100.15 ± 0.66	0.66
		16	99.52 ± 0.53	0.53	99.38 ± 0.67	0.68
Dual wavelengths	FLX	12	101.39 ± 0.33	0.33	100.55 ± 1.06	1.06
		16	99.44 ± 0.79	0.79	99.94 ± 0.75	0.75
		20	100.01 ± 0.70	0.70	99.83 ± 0.86	0.86
**Absorptivity factor**	**OLZ**	8	100.2 ± 1.89	1. 88	101.02 ± 1.65	1.64
		10	99.9 ± 0.67	0.66	99.67 ± 0.89	0.89
		12	99.56 ± 1.33	1.34	100.4 ± 1.27	1.26

^a^ The value is the mean of three determinations, SD is the standard deviation and RSD is the relative standard deviation

### Applications of the suggested methods

#### Assay of laboratory prepared mixtures

Laboratory synthetic mixtures were prepared to present the drugs in ratios that mimics that may be exist in the commercial dosage forms. Six different mixtures containing different concentration of OLZ and FLX (3:25, 2:4, 6:25, 10:20, 12:25 and 12:24 µg mL^− 1^) were prepared, Table 4. The content of either drug in each of these mixtures was obtained using the suitable proposed method. The values of % recoveries were in the range of 100.73–102.47 for OLZ and 100.40–104.47 for FLX. The values of RSD for both drugs were lower than 1.5 indicating the capability of the proposed method to find out the concentration of either drugs at extreme ratio.

**Table 4:** Determination of different mixtures of FLX and **OLZ** using dual wavelength and absorptivity factor methods, respectively

Drugs mixture (µg mL^− 1^)		Ratio	FLX (Dual wavelengths)		OLZ (Absorptivity Factor)	
OLZ	**FLX**	**OLZ: FLX**	**% Recovery** ^**a**^ **± SD**	**RSD**	**% Recovery** ^**a**^ **± SD**	**RSD**
3	25	1: 8.3	104.47 ± 0.46	0.44	102.48 ± 0.50	0.49
2	4	1: 2	100.50 ± 0.56	0.55	100.51 ± 0.80	0.79
6	25	1: 4.2	100.96 ± 0.25	0.24	101.49 ± 1.43	1.43
10	20	1: 2	101.28 ± 0.48	0.47	101.53 ± 0.65	0.64
12	25	1: 2.1	100.40 ± 0.41	0.40	100.73 ± 0.32	0.32
12	24	1: 2	101.65 ± 0.25	0.24	101.55 ± 0.28	0.28

^a^ the value is the average of three determinations

#### Assay of commercial dosage forms

The suggested methods were employed for the analysis of commercial pharmaceutical formulations containing FLX and OLZ combinations (Flunzapine 6/25 mg^®^ capsules and Raibyax 12/25 mg^®^ capsule). The found percentage recovery values were acceptable and their standard deviations were low. Thus, the proposed methods could efficiently analyze these two drugs in their combined pharmaceutical formulations. This also revealed the non-existence interference from the presence of the co-formulated excipients in the studied dosage forms. The results of the suggested methods are illustrated in Table 5. In addition, statistical comparison was performed between the obtained results with the results of reported method [5] utilizing Student`s t- and F-tests. The comparison revealed no significant variations between the compared methods as presented in Table 4. The t- and F-values obtained for all proposed methods were below the critical values at the 95% confidence level, confirming the high accuracy and precision of the examined methods.

**Table 5:** Analysis of commercial dosage form using the proposed chemometric methods and their statistical comparison with the reported method

Method	% Recovery ^a^ ± SD			
	Flunzapine 6/25 mg^®^ capsule		Raibyax 12/25 mg^®^ capsule	
	**FLX**	**OLZ**	**FLX**	**OLZ**
**Reported methods**	100.55 ± 1.25	101.47 ± 1.42	100.79 ± 0.84	100.23 ± 0.59
**Ratio subtraction**	100.34 ± 0.83 (t = 0.31, F = 2.27)^b^	100.47 ± 0.86 (t = 1.35, F = 2.73)^b^	100.84 ± 0.76 (t = 0.10, F = 1.22)^b^	100.35 ± 0.28 (t = 0.41, F = 4.44)^b^
**Dual wavelength**	101.74 ± 0.84 (t = 1.77, F = 2.21)^b^	---	100.43 ± 1.16 (t = 0.56, F = 1.91)^b^	---
**Absorptivity factor**	---	99.70 ± 0.91 (t = 2.35, F = 2.43)^b^	---	101.04 ± 0.84 (t = 1.76, F = 2.03)^b^

^a^ The value is the average of five measurements for both the proposed and reported methods

^b^ the values in parentheses are t- value and F- value. Tabulated values at 95% confidence limit are t = 2.306 and F = 6.338

### Evaluation of methods greenness

Nowadays, the implementation of green analytical procedures has become the first choice in routine drug analysis worldwide. These approaches give a guarantee that the lowest hazard to the analysts as well as the surrounding environment. Thus, the Green Analytical Chemistry achieved high popularity [29]. Various guiding rules commonly apply these fundamental concepts for wider scientific study.

Eco Score Scale is one of the metrics that has significant contributions in green analytical chemistry field [30]. This metric measures the overall adverse effects of different stages of the analytical procedure. Each stage in running the analytical methods is assigned a penalty number including the employed technique, reagents, solvents, experimental condition (such as heating, and temperature) energy consumption and waste production. These points express the hazardous impact that face the researcher during applying the intended procedure. The total number obtained was subtracted from 100 which is devoted for the ideal green procedure. The higher the final number (eco score) and closer to 100, the greener is the procedure [30]. The calculated Eco-Scale Score for the current methods was 95 owing to the absence of any extraction or heating steps, as well as the energy-consumption was very low. Table 6 summarizes the results of the eco score scale of the present method which indicated its high degree of environmental friendliness.

**Table 6:** Penalty points calculated based on Eco Scale score for the greenness evaluation of the present methods

Item	Parameter	Word sign	PP score
Technique	Spectrophotometry	LSH	1
Reagent(s)	Non		0
Solvent	0.1 N HCl (< 10 mL)	LSH	1
Heating	No heating		0
Temperature	Room temperature		0
Cooling	No cooling		0
Energy (kWh per sample)	< 10 mL		0
Waste	1–10 mL		3
Occupational hazards			0
(TPPs)			5
Eco-scale total score	= 100 - TPP		95

MSH is an abbreviation for the More severe hazard, LSH for the Less severe hazard, and TPPs for the Total penalty points

**Fig. 3:**
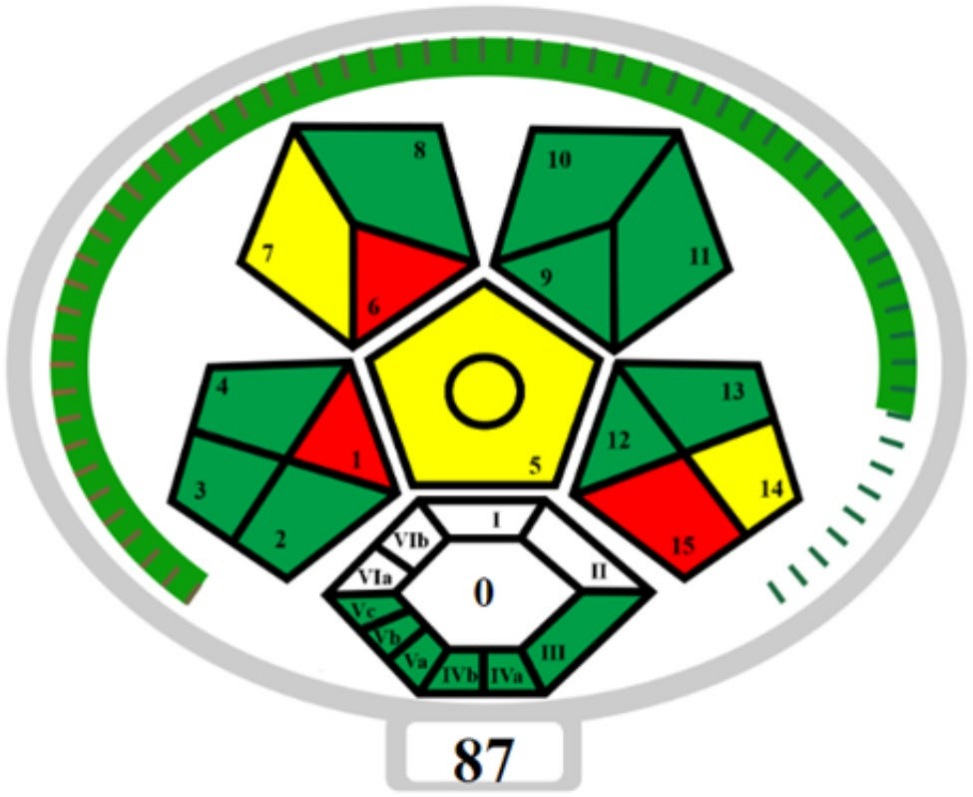
ComplexMoGAPI pictrogram of the proposed methods used for the determination of OLZ and FLX

One metric with a similar approach is GAPI, which uses five colored pentagons to rank the environmental impact of the analytical process along different steps. For enhancing GAPI, ComplexGAPI tool was proposed, including additional fields linked to the processes performed before the analyzed procedure. However, ComplexGAPI currently lacks a comprehensive scoring system for individual methods, which would facilitate easier comparisons between procedures and thus it was further improved to ComplexMoGAPI tool that integrates the visual features of ComplexGAPI with accurate total scores [31]. An associated software simplifies its application, allowing for faster and easier evaluations. The software is open source and available at bit.ly/ComplexMoGAPI. Assessment was made using three coloring codes, namely green, yellow and red in addition to a number illustrating the total scoring as depicted in. Figure 3. The output pentagrams showed that the approach presently reaches an acceptable green status as 9 areas were marked in green, 3 in yellow, and 3 in red, while the overall total score is 87.

## Conclusion

The present work was dedicated for solving the challenge of the overlapped spectra of OLZ and FLX for their concurrent, accurate and precise determination without prior physical separation. The developed methods had to venture away from those that depend on chromatography, because of its negative effects on the environment due to using hazardous organic solvents as well as their high cost. The suggested methods were based on spectrophotometry combined with simple mathematical treatments of the spectra. These approaches are simple, rapid, inexpensive, and reliable options for the effective assay of FLX and OLZ in commercial dosage forms. Ratio subtraction method was devoted for the assay of both drugs, while dual wavelength method and absorptivity factor method could be utilized for the estimation of FLX and OLZ, respectively. All the suggested methods were efficiently employed for the assay of the studied drugs in laboratory prepared mixtures and commercial dosage forms. The environmental safety of the involved procedures was assessed by applying two different green metrics. Results indicated that the methods were highly safe as no large volume of solvents were used, and no derivatizing reagent or drastic experimental conditions were involved. In addition, the procedures consume little energy and produce a small amount of waste. In conclusion, for FLX determination, the use of dual wavelength method is preferable because it needs very low mathematical treatment than the ratio subtraction method. However, for OLZ, the ratio subtraction method has higher sensitivity than absorptivity factor method while the latter is simpler.

## Data Availability

No datasets were generated or analyzed during the current study.
